# Looking at Us Through Their Eyes. The Analytical Process from Ethnographic Perspectives^1^

**DOI:** 10.1111/1468-5922.70056

**Published:** 2026-05-13

**Authors:** Stefano Carta

**Affiliations:** ^1^ Rome Italy

**Keywords:** comparative ethnopsychology, mass shooters, performative language, rituals, transgenerational processes, ethnopsychologie comparée, processus transgénérationnels, langage performatif, rituels, tueries de masse, vergleichende Ethnopsychologie, transgenerationale Prozesse, performative Sprache, Rituale, Massenmörder, etnopsicologia comparativa, processi transgenerazionali, linguaggio performativo, rituale, assassini di massa, сравнительная этнопсихология, трансгенерационные процессы, перформативный язык, ритуалы, массовые расстрелы, etnopsicología comparada, procesos transgeneracionales, lenguaje performativo, rituales, tiradores masivos, 比较民族心理学, 代际传递过程, 述行性语言, 仪式, 大规模枪击者

## Abstract

This article looks at the analytical situation through the Others’ eyes—through examples from contemporary ethnographies of foreign cultures. It discusses the following issues: a) The analogy between the ontological worlds of the dead, ghosts, animals and dreams in “primitive populations” and the analytical psychological descriptions of the unconscious; b) The way most cultures conceptualize and organize the relationship between “this” world and the “other” ones, compared with the ever‐growing Western psychology’s awareness of the role of transgenerational transmission, i.e., something that goes much beyond the importance of the one’s individual biography; c) The transferential field and process as a liminal space, within Victor Turner’s symbolic interpretation of rituals (2008) and Catherine Lutz (1988) and Alma Gottlieb’s (2004) ethnographies. Hence, the analytical process as a contemporary, modern ritual will be taken into consideration, together with transformation of the nature of language, which may shift from a referential language, to a performative one; d) Lastly, the article analyses the psycho‐social pathology of mass shooters in the USA as an archetypal emergence of a specific ritual, described by Maurice Bloch in his book *Prey into Hunter: The Politics of Religious Experience* (1991), as a perverted way to harness and control the archetypal violence that permeates our societies, especially the American one.

## Abstract (fr)

Cet article examine la situation analytique à travers les yeux des « Autres », en s'appuyant sur des exemples issus d'ethnographies contemporaines de cultures étrangères. Il aborde les points suivants: a) L'analogie entre les mondes ontologiques des morts, des fantômes, des animaux et des rêves dans les « populations primitives » et les descriptions de l'inconscient en psychologie analytique. b) La manière dont la plupart des cultures conceptualisent et organisent la relation entre « ce » monde et les « autres », comparée à la conscience croissante de la psychologie occidentale sur le rôle de la transmission transgénérationnelle — c’est‐à‐dire quelque chose qui dépasse largement l'importance de la seule biographie individuelle. À cet égard, l'article compare la position ontologique/sociale et l'identité des nouveau‐nés en Afrique (en tant que réincarnation des ancêtres) avec une théorie locale inversée de l'attachement, selon laquelle l'enfant ne doit pas être « convaincu » de devenir autonome par l'exploration, mais doit être convaincu de s'attacher à ce monde‐ci. c) Le champ et le processus transférentiel en tant qu’espace liminal, dans l’interprétation symbolique des rituels de Victor Turner (2008) et dans les ethnographies de Catherine Lutz (1988) et d’Alma Gottlieb (2004). Ainsi, le processus analytique en tant que rituel contemporain et moderne sera pris en considération, ainsi que la transformation de la nature du langage, qui peut passer d’un langage référentiel à un langage performatif. d) Enfin, l'article analyse la pathologie psychosociale des auteurs de tueries de masse (*mass shooters*) aux États‐Unis comme l'émergence archétypale d'un rituel spécifique, décrit par M. Bloch dans son livre *Prey into Hunter* (1991), comme une manière dévoyée de canaliser et de contrôler la violence archétypale qui imprègne nos sociétés, en particulier la société américaine.

## Abstract (de)

Dieser Artikel untersucht die analytische Situation aus der Perspektive der Anderen – anhand von Beispielen aus zeitgenössischen ethnographischen Studien fremder Kulturen. Er behandelt folgende Aspekte: a) die Analogie zwischen den ontologischen Welten der Toten, Geister, Tiere und Träume in "primitiven Bevölkerungsgruppen" und den analytisch psychologischen Beschreibungen des Unbewußten, b) die Art und Weise, wie die meisten Kulturen die Beziehung zwischen "dieser" Welt und den "anderen" Welten konzeptualisieren und organisieren, im Vergleich zum stetig wachsenden Bewußtsein der westlichen Psychologie für die Rolle der transgenerationalen Weitergabe – etwas, das weit über die Bedeutung der individuellen Biographie hinausgeht. In diesem Zusammenhang vergleicht der Artikel die ontologische/soziale Stellung und Identität von Neugeborenen in Afrika (als Reinkarnation der Vorfahren) und der umgekehrten lokalen Bindungstheorie, der zufolge das Kind nicht durch Erkundung zur Autonomie "überzeugt", sondern zur Bindung an diese Welt bewegt werden muß. c) Das Übertragungsfeld und der Übertragungsprozeß als Randbereich, innerhalb von Victor Turners symbolischer Interpretation von Ritualen (2008) sowie den Ethnographien von Catherine Lutz (1988) und Alma Gottlieb (2004). Daher wird der Analyseprozeß als zeitgenössisches, modernes Ritual berücksichtigt, ebenso wie der Wandel der Sprache, die sich von einer referentiellen zu einer performativen Sprache verändern kann. d) Schließlich analysiert der Artikel die psychosoziale Pathologie von Massenmördern in den USA als archetypisches Auftreten eines spezifischen Rituals, das M. Bloch in seinem Buch *Prey into Hunter* (1991) als pervertierte Methode beschreibt, die archetypische Gewalt, die unsere Gesellschaften, insbesondere die amerikanische, durchdringt, zu nutzen und zu kontrollieren.

## Abstract (it)

Questo articolo considera la situazione analitica attraverso gli occhi —con esempi provenienti da etnie straniere. Vengono qui discussi i seguenti punti: a) L'analogia tra l'ontologia dei mondi dei morti, dei fantasmi e degli animali e dei sogni nei “popoli primitivi” e le descrizioni dell'inconscio nella psicologia; b) Il modo in cui molte culture concettualizzano e organizzano la relazione tra “questo” mondo e gli “altri”, confrontato con la sempre crescente consapevolezza del ruolo della trasmissione transgenerazionale per la psicologia occidentale, qualcosa che va oltre l'importanza che ha la singola biografia individuale. A questo proposito, l'articolo mette a confronto la posizione ontologica/sociale con l'identità dei nati in Africa (come reincarnazione degli antenati) e, al contrario, la versione locale della teoria dell'attaccamento, per cui il bambino non deve essere spinto a divenire autonomo attraverso l'esplorazione, ma deve essere spinto ad attaccarsi a questo mondo; c) Il campo e il processo transferale vengono visti come uno spazio liminale, riferendosi anche all'interpretazione simbolica dei rituali di Victor Turner (2008) e Catherine Lutz (1988), e gli studi etnografici di Alma Gottlieb (2004). Perciò, il processo analitico viene visto come un rituale moderno e contemporaneo, insieme alla trasformazione della natura del linguaggio, che può spostarsi da linguaggio referenziale a performativo; d) Infine, l'articolo analizza le patologie psico‐sociali degli assassini di massa negli USA come emergenza archetipica di uno specifico rituale descritto da Bloch nel suo libro *Prey into Hunter* (1991) come modo perverso per imbrigliare e controllare la violenza archetipica che permea le nostre società, in particolare quella americana.

## Abstract (ru)

На современных этнографических примерах из зарубежных культур рассматривается аналитическая ситуация глазами Других. Обсуждаются следующие вопросы: а) Аналогия между онтологическими мирами умерших, призраков, животных и сновидений в «примитивных культурах» и в аналитических психологических описаниях бессознательного.; б) Концептуализация и организация большинством культур отношений между «этим» миром и «другими» в сравнении с постоянно растущей осознанностью западной психологией относительно роли трансгенерационной передачи, то есть выхода далеко за рамки важности индивидуальной биографии человека. С этой целью сравнивается онтологическое/социальное положение и идентичность новорожденных в Африке (как реинкарнация предков) и перевернутая с ног на голову местная теория привязанности, согласно которой ребенка не нужно «убеждать» становиться автономным благодаря исследованию мира, а необходимо убедить его привязаться/привязать себя к этому миру. в) Поле и процесс переноса как пограничное пространство в этнографических работах Виктора Тернера о символической интерпретации ритуалов (2008) и этнографических работах Кэтрин Лутц (1988) и Альмы Готлиб (2004). Аналитический процесс рассматривается как современный ритуал с учетом трансформации природы языка, который из референтного может становится перформативным. г) Наконец, в статье анализируется социально‐психологическая патология массовых расстрелов в США как архетипическое проявление специфического ритуала, описанного М. Блохом в его книге «Добыча в Охотника» (1991) как извращенный способ обуздания и регуляции архетипического насилия, которое пронизывает наши общества, особенно американское.

## Abstract (es)

Este artículo analiza la situación analítica a través de los ojos de los Otros, mediante ejemplos de etnografías contemporáneas de culturas extranjeras. Aborda las siguientes cuestiones: a) La analogía entre los mundos ontológicos de los muertos, los fantasmas, los animales y los sueños en las “poblaciones primitivas” y las descripciones del inconsciente desde la psicología analítica; b) La forma en que la mayoría de las culturas conceptualizan y organizan la relación entre “este” mundo y los “otros”, en comparación con la creciente conciencia de la psicología Occidental sobre el papel de la transmisión transgeneracional, es decir, algo que va mucho más allá de la importancia de la biografía individual. En este sentido, el artículo compara la posición ontológica/social y la identidad de los recién nacidos en África (como reencarnación de los antepasados) y la teoría local invertida de la teoría del apego, según la cual no hay que “convencer” al niño de que se vuelva autónomo a través de la exploración, sino que hay que convencerlo de que se apegue a este mundo. c) El campo y el proceso transferencial como espacio liminal, dentro de la interpretación simbólica de los rituales de Victor Turner (2008) y las etnografías de Catherine Lutz (1988) y Alma Gottlieb (2004). Por lo tanto, se tendrá en cuenta el proceso analítico como un ritual contemporáneo y moderno, junto con la transformación de la naturaleza del lenguaje, que puede pasar de ser un lenguaje referencial a uno performativo. d) Por último, el artículo analiza la patología psicosocial de los tiradores masivos en los Estados Unidos como un emergente arquetípico de un ritual específico, descrito por M. Bloch en su libro *Prey into Hunter* (1991), como una forma pervertida de aprovechar y controlar la violencia arquetípica que permea nuestras sociedades, especialmente la estadounidense.

## Abstract (zh)

通过他者之眼观照自身:民族志视角下的分析过程本文通过当代异文化民族志案例, 以“他者”视角审视分析情境, 探讨以下议题:a)“原始族群”关于亡者、幽灵、动物与梦境的本体论世界, 与分析心理学对无意识描述之间的类比关系；b)多数文化对“这个世界”与“他者世界”关系的概念化与组织方式, 与西方心理学日益关注的代际传递现象 (其意义远超个体生命史范畴)之间的对照。在此维度上, 本文比较了非洲新生儿作为祖先转世的本体/社会身份定位, 与当地颠倒的依恋理论——该理论主张不应劝导儿童通过探索实现自主, 而应引导其主动“依附”于这个世界。c)借维克多·特纳的仪式象征理论(2008)及凯瑟琳·卢茨(1988)、阿尔玛·戈特利布(2004)的民族志研究, 将移情场域与过程解读为阈限空间。由此, 分析过程作为一种现代仪式将被重新考量, 同时, 也探讨了语言特性的转变, 从指涉性语言转向为述行性语言。d)最后, 文章分析了M·布洛赫《从猎物到猎手》(1991)中描述的美国大规模枪击案中的社会心理病理, 把它理解为一种的特定仪式的原型性涌现, 这是一种这种扭曲的, 对渗透社会 (尤以美国为甚)的原型暴力进行驾驭和操控的方法。

*If we look back into the past history of mankind, we find, among many other religious convictions, a universal belief in the existence of phantoms or ethereal beings who dwell in the neighbourhood of men and who exercise an invisible yet powerful influence upon them. These beings are generally supposed to be the spirits or souls of the dead. This belief is to be found among highly civilized peoples as well as among Australian aborigines, who are still living in the Stone Age. Among Western peoples, however, belief in spirits has been counteracted by the rationalism and scientific enlightenment of the last one hundred and fifty years, so that among the majority of educated people today it has been suppressed along with other metaphysical beliefs*. (Jung, 1988, para. 570)

In his masterful essay “The uncanny” (2003), Freud describes the displacing experience of seeing himself as a wholly‐Other. I will try to take a similar perspective and look at our analytical situation through the Others’ eyes—through discussing examples from contemporary ethnographies of foreign cultures. I believe that such a mirroring may help us to better understand what we ourselves do and think.

I would like to take you through a visit to a gallery in whose rooms I will always juxtapose two series of scenes and considerations, one coming from ethnography and the other from the analytical situation.

## The First Room: What is a Real World?

Insofar as one must always have a point of departure, a perspective, through which one looks, it is impossible not to be ethnocentric. What one can and should do is to be *critically* ethnocentric. I am skeptical of Jung’s consideration that modern psychology offers a more sophisticated or advanced approach to the soul than other cultural traditions throughout the world’s history. Along with Edward Evans‐Pritchard, I do not believe in savages, in people who believe (them) versus those who think (us), but, along with Lévi‐Strauss I do believe in primitive layers of the human mind. In fact, when I take the analytical perspective seriously, I often find myself taking on the *Others’ perspective* (may that sometimes be those of a shaman, of a diviner, or a seer).

These other perspectives are of an under‐upper world made at the same time of dreams and wakeful perceptions; a multiverse inhabited by spirits, ancestors apparently dead long ago, and talking animals whose presence and actions reveal deep psychological, spiritual, and instinctual truths, together with the barman that serves my coffee every morning, my wife and children, my colleagues that I meet every day at work.

Each one of these subjects, those from *this* world and those from the other ones, are equally real. As William James and Jung thought, they are real whenever they are emotionally effective. To make myself clearer, I will show the first two pictures – two short clinical vignettes.

Franca was a 28‐year‐old Italian woman. Her childhood and youth had been traumatic. She was an unwanted only child. When Franca was one year old her mother gave her to her own mother, in her own attempt to win her love, which she had missed all along. Franca stayed with her parents only for Sunday lunch. When Franca was five years old her mother reclaimed her without any consideration for her feelings towards her grandmother. Her mother just “needed her”. Her father was violent and verbally abusive. It is enough to say that he would play Russian roulette with Franca when she was a child. She had no idea that the gun was not loaded.

Franca was a truly wonderful soul. She was sensitive and very much beloved by everyone, including her working colleagues and boss. Since the first session, she would sit in from of me and silently cry and cry and cry slow warm tears. From the beginning of our work together I had felt that she was special. She was capable of love, care, compassion, and had a deep ethical nature. After two years she travelled to India. She returned overwhelmed. She loved it; she connected with the people; she bought herself a sari and loved to dress in it. She now was like an enchanted child who could finally dream of something beautiful. Then this dream arrived:

I am with my colleagues and my boss. I feel somehow agitated. They are all sitting in a circle. My boss is standing up. I am next to him. There is an empty chair. My boss says: “Franca, will you sit on this chair?” I say yes and sit down. Again, I feel somehow anxious. Now, my boss says to me: “Franca, will you let me blindfold you?” I am very surprised and quite alarmed. I think that if I let him blindfold me, I will lose all control and I will be at the mercy of anything. Yet, I trust him and I let him blindfold me. Now I am blinded. The silence is perfect. There is a growing tension. Then I perceive something approaching me. I am holding my breath when an Indian woman suddenly lifts the blindfold. She is standing right in front of me, staring right into my eyes. She is wearing a long sari. She is beautiful and intense and starts speaking to me in a language foreign to me. Suddenly, from this unknown language a sentence emerges that I perfectly understand. Looking straight in my eyes, she forcefully tells me: “I, am your mother”.

This dream had the most dramatic of consequences. After it, Franca has never been the same.

Now, thinking of Franca, and although this may sound factually crazy, I am ready to reverse the normal order of things and, as an Amerindian medicine man, I have no hesitation to say that what we may refer to Franca’s real world—the biographical world of her family, of her youth—was not really real, and that who she really was was the Indian’s woman’s daughter, the daughter of a spirit.

As Plato would have easily recognized, Franca’s soul had incarnated in the wrong world. The anthropologist Tobie Nathan rightly says that a symptom is a text without a context (Nathan, 2018). In this case I would rephrase this and say that a symptomatic existence is like a text embedded in a *wrong* context. In effect, I am convinced that in our analytical practice we do not have to try to transform or change the text, the patient, but we must search for the right context in which the patient’s story actually *makes sense*.

Perhaps, some of us would suggest using these conclusions only as metaphors, so to maintain that our cosmology is real while other ones are merely poetic fantasies. Then, we would say that Franca’s dream image was a fiction of her mind, a mere as‐if, because Franca’s parents were obviously those of flesh and bone.

I do not agree. I think we must consider all possible ontological perspectives as equally real, as long as they meaningfully resonate with the person’s nature. Very often I listen to what my patients tell me about their daily businesses, about the *samsara* of their daily lives as if they were dreams, and often it is by this turning of facts into dreams that I begin to unravel these mute and blind facts that “just happened to them” and in that unraveling, find the meaningful experience. In a way, facts are dreams in disguise that are trying to enter into the ego’s daily life.

Now, a second case.

Jenny was a 32‐year‐old Liberian refugee living in Rome. Jenny’s story was dreadful. Most of her relatives were killed in the savage civil war taking place in her country. She was forced into prostitution and raped multiple times and decided to flee Liberia to avoid being killed. She reached Italy through a brutal, endless voyage across Africa. Jenny was referred by the public psychiatric service to a colleague who I was supervising; her diagnosis: paranoid schizophrenia. She was infested by several Djinns that would fill her mind with auditory hallucinations, giving her contradictory orders, warnings and enigmatic interpretations.

For the psychiatric services the etiology was clear: Jenny’s mind had collapsed because of her multiple, repeated, complex traumatic experiences. The psychiatrists had prescribed massive doses of antipsychotic medications but Jenny was a so‐called “non‐respondent”. The drugs had no effect whatsoever on her schizophrenic symptoms.

She very slowly developed a relationship of trust with her analyst, which made it possible for her to eventually reveal her secret. We must remember that often there are secrets that our patients keep to defend themselves from their therapists. This is quite frequent with patients who come to us from foreign worlds. These secrets are often the crucial element without which nothing therapeutic may happen.

For Jenny, the breakthrough was the *confessio* of her own secret: she belonged to a matrilinear lineage in which, in every generation, one woman was destined to be a healer, and Jenny was the chosen one. She had completely disavowed this destiny and refused to go through the necessary rituals to become a medicine‐woman herself. To make a long and difficult story short, eventually Jenny returned to Liberia and was initiated into her destined path. Then, she returned to Rome. What I can say is that her Djinns decreased and that they slowly interacted in a more civil manner with her and among themselves.

Jenny remained a liminal being, perhaps a bit like a shaman, but she was no longer overwhelmed by her spirits and could carry on a decent life. She also became an influential person among her group, as she was recognized by many as an expert on the world of spirits.

Why was Jenny not responsive to antipsychotic drugs? And why could Franca be transformed by her dream, a dream that still moves me so much, after almost thirty years? Very simply put, Jenny was not responding because, unknowingly and with the best of their intentions, the psychiatrists were not really treating her; they were treating the projection of their own western modern cosmological world, the one Philippe Descola (2013) calls *naturalism*, for which the protagonist and the subject is the *material* body. For Jenny’s animistic cosmology, the attempts of our Western naturalistic psychiatrists, who lived in a universe made of material bodies and not of spiritual presences were—so to say—like those of a tailor who tries to heal back pain by ironing the patient’s shirt.

The failure of the psychiatrists’ naturalistic approach would also have made no sense for the other three cosmologies that Descola individuated: animism, totemism and analogism. In fact:

While animism sees signs of otherness in the discontinuity of bodies, naturalism finds them in the discontinuity of minds. I am different from someone who, speaking another language, believing in other values, thinking according to other categories, and seeing things according to another “worldview,” is no longer just like me because the “collective representations” to which he adheres and that condition his actions are so very different from mine. […] For naturalist subjects, there is unfortunately no mental equivalent to metamorphosis; all we have at our disposal are the unsuccessful attempts made by poetry, psychoanalysis, or mysticism. In these circumstances, it is understandable that radical otherness seems to lie on the side of those either devoid of minds or who do not know how to use them: savages (in the past), the mentally ill (today), and, above all, the immense multitude of nonhumans, animals, objects, plants, stones, clouds, all this material chaos that exists in a mechanical fashion and with laws of composition and functioning that humans, in their wisdom, work busily to discover. (Descola, 2013, p. 143)

In passing, I would like to point out the fact that the naturalistic idea of a unique bodily nature not only risks reducing the polymorphous nature of the psyche to the biological body, but also that it always refers to the body as it is politically construed and conceptualized by the dominant class.

In times of gender fluidity, the decoupling of the naturalistic biological sex from the multiplicity of the spirit seems to me a sign of a seismic ontological shift, which I welcome.

On the other hand, in a perspectivist animistic cosmology:

The diversity of the classificatory indicators used by Amerindians to account for the relations between organisms shows just how flexible boundaries are in the taxonomy of living beings. For the characteristics attributed to the entities that people the cosmos depend not so much on a prior definition of their essence but rather on the positions that they occupy in relation to one another by reason of the needs of their metabolism and, in particular, their diet [I will go back to this issue in the last room]. The identities of human beings, both living and dead, and of plants, animals, and spirits are altogether relational and are therefore subject to mutations and metamorphoses depending on the point of view adopted. In many cases it is said that an individual of one species apprehends the members of other species in accordance with his own criteria, so that, in normal conditions, a hunter will not realize that his animal‐prey sees itself as a human being, or that it sees the hunter as a jaguar. Similarly, a jaguar regards the blood that it drinks as manioc beer, while the monkey‐spider that the cacique bird thinks it is hunting is, to a man, nothing but a grasshopper, and the tapirs that a snake considers as its preferred prey are really human beings. It is thanks to the ongoing swapping of appearances engendered by these shifting perspectives that animals in all good faith consider themselves endowed with the same cultural attributes as human beings.^2^ (Descola, 2013, p. 18)

The entire etiological theory to which Jenny’s psychiatrists adhered was a narrative completely foreign to her. It was the reproduction of a fashionable theory of trauma shared by most *scientifically* trained psychologists, in this case a naturalistic reproduction forcefully imposed on Jenny, colonizing her own secretly kept cosmology.

In fact, for most of us a trauma must be the effect of something that happened in our life, in our biography, in *this* material world, because we honestly think, as Franca thought until her revelatory dream, that we *are* our biography and that it is to *this* world that we belong—and to where, otherwise?

Let’s move to the second room and think about it together.

## The Second Room. Being Born: Who is the Baby?

In our Western world and in our Western psychology we believe that a newborn is endowed with a specific, natural motivational system — attachment — and that we must support it to promote what we call *exploration*, an activity that springs from a second motivational system that Jaak Panksepp called *Seeking*. Hence, our image is that of a baby who is *naturally* attached to this world—provided that the Great Mother archetype projected onto the caregiver holds such attachment and supports the natural tendency of the newborn to become a whole person through a separation‐individuation process.

But is this a universal point of view?

When, in most African societies, a baby is born, the women who witness the birth, together with seers and elders, try to figure out who the baby is. They do this starting from their *first impressions*, which will be further elaborated and explored through careful inspection, rituals and divination procedures. This happens because the newborn is the incarnation of a spirit, or the reincarnation of an ancestor.

Will the response of the search for the baby’s complex identity confirm those first impressions and those first feelings? Eventually, the baby’s body and behaviour will say which ancestor comes to resume its place among the living.

Thus, within this animistic ontology a father may recognize in the baby his own father and call him “Dad”.

In my opinion this vision is very interesting and makes very much sense, because appropriate rituals—providing a pragmatic, symbolic structure made of multiple connections that involve the social sphere in which the baby is about to be included—immediately thematizes the fact that all babies are the recipients of what we analysts may call *transgenerational projections*.

In these societies, although from the time of one’s birth one already carries a kind of bond with the world of the dead, it is important to understand that these bonds do not abolish the individual; they do not establish a kind of being without personal, idiosyncratic characters, a sort of a diffuse subject wholly defined by his or her social belonging. On the contrary, systematic attention and efforts are given to the *differentiation* of individual identities through the differentiation of roles, of economic and professional classes and marriage rules, together with the distinction of age classes, initiation periods, mourning rituals, and so on. All these components will define a specific individual as a unique emergent being within a complex social network that links the living and the dead through transgenerational time (Beneduce, 2007).

I suppose that the fact that the divination of the baby’s nature starts from its elders’ first impressions points to the fact that the entire process is based on what Jung called *intuition*—the quality of the unconscious’ consciousness—something that we all know provides us exceedingly important information about our patients during the first minutes and hours of the first sessions, when we do not yet think to know them.

Hence, differently from our perspective based on attachment, in these cultures – in most of the non‐Western cultures the baby must be *convinced* to attach himself to the world of the living—to his own social world—and therefore *detach* himself from the world of the dead. This is done by negotiating with the ancestor or the spirit that is trying to enter this world with him.

Differently from ours, this animistic perspective is based on and fosters a profound vision of the person as deeply culturally embedded, and with “culturally” I include the complex network between the ancestors, the spirits, the animals (who have a key role in the totemic ontologies) and, of course, the living, who are promoting the full birth of this hybrid, liminal being that is the newborn. Therefore, unlike in our atomized naturalistic societies, these cultures interpret the world as an ecological system of relations based on complex transmissions at every level.

Hence, for instance, through these complex rituals, the baby will be given a name. Catherine Lutz writes that for the Ifaluk of Micronesia

The act of name giving is seen as an important statement of connection with the infant, and people will often give accounts of the origins of their names. The linguistic label itself is always a unique one, as a name can never be that of an ancestor, for to mention the name of an ancestor is to call forth his or her spirit. This syllable, however, may be taken from the name of the different ancestor. Thus, naming is seen as creating a bond between the child and both the creator of the name and the ancestors whose names were in part used. (Lutz, 1988, p. 106)

Or, according to Marilyn Strathern,

The Hagen [of New Guinea] view of the person, by contrast, does not require that a child be trained into social adulthood from a presocial state, nor postulate that each of us must repeat the original domestication of humanity to deal with the elements of a precultural nature. Society is not a set of controls over and against the individual; human accomplishments do not culminate in culture. (Strathern, 1988, p. 89)

This perspective, according to Marshall Sahlins (2008), demolishes the claim of universality of Freud’s theory of a “natural” bestial Id and of a repressive culturalizing Superego. Rane Willerslev (2007), for example, notes that for the Yukaghir of Eastern Siberia “children do not exist,” since for them children have the skills, knowledge, temperament and attributes of the dead family members whose reincarnation they are. They are cultural beings *from the start*. Many of these characteristics are forgotten when the child learns to speak and are gradually recovered over the course of the child's life. In a work entitled *The Afterlife is Where We Come From* (2004), Alma Gottlieb describes the functionally analogous idea of the Ivory Coast Beng: the child only gradually manifests the personality of the family member he incarnates because the other dead try to keep him with them.

Not only does this view of what a baby is promote a profound integration at all levels—social, cultural, symbolic and spiritual—hence protecting the individual from falling into the utter desperation of isolation, it also has a fundamental prophylactic function for the innumerable cases that we see in our own practice, where patients of all ages are faced with the same task of recognizing which spirit, which dead relative, or which unsolved transgenerational problem has infested their lives but is yet unseen.

Here is one of the many examples I could give you.

Laura is a forty‐five‐year‐old woman. She had been in a second analysis with me for two years. Laura was managing to separate from her extremely manipulative, gaslighting husband. She was trying to slowly build her own autonomy. Despite her talents and postgraduate education, Laura had never worked because she had always supported herself through an important inheritance left to her by her maternal grandfather. Laura had a very complicated relationship with her extremely intrusive, self‐centered, narcissistic incestuous mother, Lucia. Her father died when she was eight years old and was remembered as a sweet, reliable, mythical figure, unfortunately lost to her.

One night, in the week of her birthday, Laura had a dream in which she sees a spiral. This dream left her surprised and disturbed. During the session after the dream, she starts talking about her 13‐year‐old daughter Rossella (note that both daughters were molested by a male babysitter of sorts, with immense guilt on Laura’s part). Rossella kept saying that her mind was busy and that it was impossible to concentrate. She was suffering from fits of depressive moods and rage and could not study. She told Laura that she wanted to die or run away from home. I tell Laura that Rossella’s block seems to me somehow similar to her own block having never been worked out, something like a transgenerational issue. At this point, Laura, somehow puzzled, tells me that, by the way, her great‐grandmother Carla had been born exactly 100 years before her, and she recounts the story.

Laura’s maternal grandfather, whom I will call Maurizio, had always been extremely close to his own mother Anna, Laura’s great‐grandmother—an ambitious woman who had been culturally castrated by the Italian patriarchal culture.

Maurizio seems in some ways a puer, the son of the Great Mother. He lived the heroic life of a conquistador and perhaps did so for his mother. From a condition of deep poverty, Maurizio became very, very rich. Maurizio fell in love and married Laura’s grandmother, Anna. She had a high school diploma and would have liked to keep on studying and graduating, but she could not do it, partly because Maurizio, completely immersed in his heroic mission that would lead him to become economically extremely successful, forced her to the confining life of mother and wife.

Out of this relationship Laura’s mother, Lucia, was born. She had a very intense and ambivalent relationship with her father Maurizio. When, still very young, she got pregnant with Laura, she got married in protest against her monumental father—Maurizio, Laura's grandfather. Perhaps she did it because he never had been a father but had remained a puer‐hero, who did not know how to give fatherly love to his children, but knew only to compete to redeem the archetypal wound of his mother—Carla, Laura’s great‐grandmother.

When Laura was a child, her mother demanded that she study piano and cello ‐i.e., to do and be what neither she, nor her own mother or grandmother, Anna, had been able to be. At his death, Laura's grandfather Maurizio left Laura his inheritance, repudiating and bypassing Lucia, Laura’s mother. The consequence was the creation of a terrible transgenerational triangulation. At the same time, Laura’s mother, Lucia, was taking over the life of the young Laura, not only through music‐related constraints but, when Laura was a teenager, even by sexually seducing her first 16‐year‐old lover. Laura’s mother would fit well the description of the devouring semi‐psychotic witch.

In the session, Laura now connects her daughter Rossella’s fits when, at Rossella’s age, she used to cut her own chest, or bite herself, or when she used to scratch her cello. She recalls that for her, the cello and music study had no meaning and how they were intrusively imposed by Lucia, her mother. In the following weeks the dark nature of her grandfather Maurizio’s inheritance becomes clear: it had not been a resource, but a spell. As the sessions continued, her immense guilt for the fact that both of her daughters had been molested slowly lifts as she recognizes that she herself had gone through similar ordeals.

Laura was eventually able to free herself from her husband. In the meanwhile, in the following weeks Rossella suddenly got unblocked, re‐acquired her serenity and was able to do her homework with no further problem whatsoever.

With Laura, we are dealing with the transgenerational transition of a wounded and simultaneously ambitious animus, which fails to integrate with the personalities of these women at every generational transition. This animus didn’t find in any father the necessary ferryman to transform its archetypal mode to become a resource for the ego. It was a paralyzed animus, gradually projected on each successive generation.

In a traditional context Laura would have been ritually asked which spirit she was already embodying at her birth. Unfortunately, this had to wait for over forty years of an unhappy and sometimes even tragic life, until she could find the question ‐ and its answer ‐ in our analysis.

It is apparent that the spirit that should have been evoked and negotiated with at Laura's birth was her great‐grandmother, born exactly one hundred years before her.

We know that attachment theory is transculturally quite well confirmed. Therefore, *both perspectives seem to be valid and useful*, while at the same time deeply culturally bound. I suppose that attachment promotes the recognition and the interpretation of the coming into existence of a human being referring to his *biography as a separate, new individual*. The traditional view interprets the same process through the recognition of a person’s *mythobiograpy*; of a being that is from his very origin deeply embedded in a multiverse made of complex relational networks. This was, for instance, the case of Jenny that I spoke of before. While our scientistic, naturalistic view, pointing to one’s biography, takes into consideration the personal unconscious, the traditional mythobiographical perspective takes into consideration the cultural unconscious and the collective, archetypal unconscious.

Therefore, what we Western analysts call “interpretation” when—inspired by attachment or post‐Freudian theories—we refer to the patient’s biographical youth, perhaps corresponds to the process of *divination* that takes place when the newborn is recognized as a subject who conveys transgenerational and spiritual meanings and issues still relevant for his family, if not for the whole social group.

The question now is: When, and in what conditions, do interpretations and divinations work? Let’s move to the next room and see.

## The Third Room: Liminality, Speaking Words, Making Reality

In the previous room of our gallery we have seen that the process through which a person is born is a ritual process. As you will see in the fourth room, the issue of rituals is a central point of my presentation, because I think the analytical process is one of the very few means left in which to perform effective rituals of becoming oneself in our atomized, naturalistic (Western) world.

In this third room I find myself asking myself a question: Thinking about the role that language plays in analysis, what is it that really makes the statement an analyst utters *work* for the patient? Is it an interpretation? An amplification? To respond to this question, I turn to the linguist John Austin (2020) who distinguished two kinds of statements: the first kind is made of constative statements, which express something that (and here I am simplifying) may be either true or false: *Yesterday it rained a lot* and *My father’s name is George* are examples of such statements.

The second kind of statement is what interests us and describes what Austin called *performatives*. A performative statement does not just say something about something else, it does not necessarily *refer* something to something else in a way that may be true or false; a performative statement is linguistic *act ‐* it *makes* the thing it says by saying it: *The meeting is adjourned*; *I hereby declare you husband and wife*; and perhaps also *Your foetus is female (or male)*. All these sentences correspond to the action they describe; this action is performed precisely because it is said.

According to some anthropologists, such as Benjamin Ray (1973), Godfrey Lienhardt (1961), Ruth Finnigan, Genevieve Calame‐Griaule (1965), and Marcel Griaule (1965) all rituals work through the use of performatives, as magic spells do and as, in my opinion, does analysis. Ray uses as evidence for his performative account of language in rituals, which he regards as its central mechanism, instances in ritual language in Dinka and Dogon religious practices. He asserts that when the Dinka from Sudan perform animal sacrifices for the recovery of a sick tribesmen, the sentences spoken in the ritual by their very utterance achieve culturally and socially defined objectives of various sorts. For example, in a sacrificial ceremony the words said are meant to *establish* states of affairs, and, in fact, bring them about. Ray supports his claim by showing how the Dinka priest performing the ritual not only *refers* to his act of separating the spirit that is causing the illness from the body of the sick, but also thereby *accomplishes* this task through the force of his verbal command.

Remember my opening question about what really works in analysis when the analyst says something. If I look at the analytical situation from the anthropological perspective, I realize that, while they fall into the category of constantive statements, interpretations and amplifications perhaps are not what really counts. They do not produce any true psychological transformation. Rather, interpretations and amplifications as such refer to a central domain different from performatives and common to both rituals and analysis – something we are very familiar with – the *symbolic domain*.

Through a good interpretation or a productive amplification, a symbolic meaning may coalesce that will refer to the criterion of truthfulness or falseness. I find this symbolic elaboration is a necessary *precondition* for analysis to work, but it is not sufficient for a transformation to take place.

The long thread of interpretations and amplifications that the analyst offers session after session, her devotion to understanding or her openness to new potentially true meanings to emerge seem to me merely conditions, although necessary conditions, for the transformation of the nature of language from constantive to performative, without which nothing really takes place.

Along Northrop Frye (1984), I suppose that the deep relationship between the symbolic word and the performative word may be traced in the affinity between the two Greek words: “*symbolon”* and “*symbolos”*. Symbolon, a noun of neutral gender is the signifier that Jung described – an *indication towards* a potential “true meaning” —while symbolos, a masculine noun means “omen”, “augury”, i.e., the epiphany of an actualized, present *portent*.

Let’s take the case of an interpretation. For 99 times I may tell a patient: “You have been really living in a painful loneliness”. He might recognize the statement as true, but nothing would actually *happen*. In fact, for a transformation to occur, any constantive statement must become performative. *This* is the necessary condition by which language may work for the analytical goal of the promotion of the patient’s individuation. This may happen perhaps the hundredth time I will tell him the same thing. Only then he will *realize* that actually, *Yes!* This is *true! This is who I am!* (This issue is also discussed by Winnicott, 1961).

In this case, the analyst’s statement is not true because he was *referring* to something in a true way, but because his language shifted towards the same cosmogonic quality of God’s language when He uttered the sentence: “*Let there be light*”.

At his 1967 Harvard Lectures Jorge Luis Borges said:

[…] words began not by being abstract, but rather by being concrete, and concrete, I suppose, would be the same as poetic. […] it seems obvious to me that words began, in a sense, as magic. Perhaps, there was a moment when the word “light” seemed to be flashing, and when the world “night” was a dark word. (Borges, 1967‐1968)

Performatives seem to reconnect to this primordial, magical condition in which saying is *really* creating. This is the language of fullness, the Mother’s word that is uttered with the object present, not the Father’s word that represents the object lost. This creative Logos, therefore, is not abstractly spiritual but is connected to our empirical, experiential original truths based on perceptions and actions imbued with meaning and scope. I think that words become traumatic not when they refer to something violent—this condition is necessary, but not sufficient. They become traumatic when their violent meaning makes words shift into the performative realm.

Performatives are creative because, as Winnicott may say, they take a potential into existence. In analysis they make it possible to formulate the only possibly correct answer to one of the fundamental, structural analytical questions posed by the mirroring analyst and to which the patient has to respond: *Who are you?* As you will remember, this was the first, seminal question posed to the newborn within the traditional ritual situation.

The performative nature of utterances does not refer to their grammatical features; *any* statement may become a performative statement. Yet, this may happen only in certain conditions that match both the rituals as studied by anthropology and the analytic situation.

In Table 1, I summarize these conditions:

**Table 1:** Comparison of the general conditions for performative utterances. In square brackets the analytical situation’s correspondences with the ritual’s conditions (as in Austin, 2020, p. 19) with the analytical relationship and setting (Austin, 2020, p. 14).

(A. 1) There must exist an accepted conventional procedure [*Securing an analytical liturgy*] having a certain conventional effect, that procedure to include the uttering of certain words by certain persons [*Projecting the archetypal function of the healer on the analyst*] in certain circumstances, [*Promoting an analytical setting*] and further, (A. 2) the particular persons and circumstances in a given case must be appropriate for the invocation of the particular procedure invoked [*Reciprocal recognition of the analyst‐patient play*]. (B. 1) The procedure must be executed by all participants both correctly and (B. 2) completely *[“Alchemical commitment” to the analytical* opus] (Г. 1) Where, as often, the procedure is designed for use by persons having certain thoughts or feelings, or for the inauguration of certain consequential conduct on the part of any participant, then a person participating in and so invoking the procedure must in fact have those thoughts or feelings, and the participants must intend so to conduct themselves, [*Essential empathetic and ethical mirroring disposition of the analyst when he “interprets”*] and, further they: (Г. 2) must actually so conduct themselves subsequently.

I would like to underscore that the realm of performatives, which *are pragmatic actions*, overlaps the Adlerian stage of analysis that Jung calls “education”. In fact, mere “elucidation”—understanding through constantive, symbolic language—through interpretation or amplification—is far from being enough, as for the realization of an understanding to occur it must resolve into an *empirical truth*; *a truth that produces some interpersonal and social effect*.

Consciousness does not limit itself to cognitive understanding. It has an empirical aspect for which a “realization” acquires both its two meanings—“understanding” and “making real”—through our own conduct. This performative aspect of language, which has to do with the alchemical *rubedo*, is for me very important, as it is the base of my own personal involvement in the activity of the group of “Analysis and Activism” within the IAAP.

Now, there is another essential element that must be taken into consideration. The transformation of cognition into experience can happen only when the statement is endowed with what Austin (2020) calls illocutionary *force* (after the logician Gottlieb Frege, 1918); or what I would call *numinosity*, or *affectivity*. It is when a statement, a word, suddenly begins to express and emanate affectivity, numinosity—force—that language is about to shift. Jung researched such a *force* during his experiments with word associations. It is when a word carries an intense affect that saying it *makes real something of the subject’s self*.

When the analyst’s language becomes experientially effective and endowed with that cosmogonic quality by which the potential self enters into existence, the analytical situation finally becomes what it should be—a religious ritual—and, therefore, it acquires a true transformative effect.

If we look at what we do through the symbolic perspective and take into consideration, for example, Victor Turner’s ethnography of the Ndmbu of Zambia (2008), we may appreciate the profound connection between the movement that rituals organize from what Turner calls a “structure” to an “anti‐structure”, and then back to structure. Turner’s theory is inspired by Van Gennep’s model of the rites of passage (1961), of which I will speak in the last room of this gallery. For both van Gennep and Turner, between the *rite d’entrée and the rite de sortie* there is a liminal space, in which a transformation is about to occur.

For us analysts this liminal area is fundamental, because it describes the nature of co‐transference, in which it is not the consciousnesses of analyst and patient that talk to each other, but rather where their unconsciousnesses enter into dialogue. As Jeffrey Miller pointed out (2004) the liminal phase de‐structures the patient’s persona and, I think, also that of the analyst, insofar as he is available to fully commit to the analytical process.

Within this liminal territory, the unconsciousness of analyst and patient become partially fused and — transitioning from a structure to an anti‐structure—they may eventually meet precisely through an undoing some part of what they believe to be in order to become who they must become: conscious of their own projections. Such an anti‐structuring phase, this liminal space in which structures turn into anti‐structures marks the beginning of a *nigredo*. It is fundamentally a land of dreams and fusional affectivity.

It is within this space, the liminal space of co‐transference, that the shift from a constantive, propositional language to a performative one may take place. In this space, like in dreams, what is imagined by both and said by the analyst may become really real.

## The Fourth Room: The Loss of Rituals and the Task of Analysis

This last room is a dark room that allows me to connect, once again, with the psychosocial dimension of the soul. I started this presentation with birth and I will finish with death—with violent, anomic death—because I wish to look at the terrible phenomenon of mass shootings in the United States to discuss the lack of rituals in modernity. Although this phenomenon is typical of the United States, it is the indicator of the general crisis of our neoliberal, capitalist societies which in the United States appears in this extreme way.

The phenomenon of the shootings may be connected with the critical state of our Western ontology. As early as 1938, sociologist Robert Merton argued that the very structure of American society pressured people to commit crimes—people who failed to achieve what they were socialized to believe was their destiny, who found their aspirations for status and wealth blocked and were forced to adapt.

More than in other Western countries, American citizens, especially those that have felt entitled to pursue and gain the happiness promised in the American Declaration of Independence—in particular white males—seem to be exposed to an unbearable feeling that the literary critic Lauren Berlant (2011) calls “cruel optimism”:

A relation of cruel optimism exists when something you desire is actually an obstacle to your flourishing. It might involve food, or a kind of love; it might be a fantasy of the good life, or a political project. It might rest on something simpler, too, like a new habit that promises to induce in you an improved way of being. These kinds of optimistic relation are not inherently cruel. They become cruel only when the object that draws your attachment actively impedes the aim that brought you to it initially. (p. 9)

[…] optimism is cruel when the object/scene that ignites a sense of possibility actually makes it impossible to attain the expansive transformation for which a person or a people risks striving; and, doubly, it is cruel insofar as the very pleasures of being inside a relation have become sustaining regardless of the content of the relation, such that a person or a world finds itself bound to a situation of profound threat that is, at the same time, profoundly confirming. (p. 10)

What I wish to underscore here is the sense of shame and humiliation produced by such an increasingly anomic situation. Together with the well‐researched relationship between humiliation and violence—both against the self and against others—another very interesting feature is the tilting of a *depressive* sense of shameful powerlessness into a *paranoid* violent reaction—may that be suicide or homicide—ultimately aimed at protecting Winnicott’s true self from being reduced to a humiliated, degraded object.

In the crisis of the West, the overflowing subject, whose icon is the self‐made man (a typical characteristic of the Gnostic Archons, the masters of this lower world, who think to be God), can no longer bear the sense of individual responsibility which now demands of him something impossible—to now take responsibility not for his own success, but for his own existential failure, his humiliated condition.

This extreme anomic situation could be resolved through a profound transformation of the cultural complexes, of the ethos of a nation if not of the whole West, something that the poor lonely subject, especially those belonging precisely to the ranks of the “weakest” (the “losers”) cannot by himself pursue, nor even conceive of.

This situation produces an enantiodromia that from the mania‐depression axis typical of a psyche that feels omnipotently responsible for its own deeds and thoughts, flips to the paranoic state of a psyche that feels attacked and endangered by enigmatic sources. When depression turns to paranoia the risk will then be not only of suicide, but of the hatred of any object upon which it is possible to project our persecutory feelings: a political opponent, the colour of another’s skin, some different gender.

Andras Zempleni gave an important contribution to just this issue of the shift in psychopathology due to the traumatic process of urbanization in Africa. One example was the Bregbo community of the Ivory Coast, where a healer‐prophet, Atcho, promoted public confession sessions. (Here my thoughts turn to American television preachers, a phenomenon truly astounding for its psychotic‐archetypal evidence of the profound nostalgia for anything religious, in which the only god left is the Biblical, capitalist Mammon: Money.) Zempleni interprets these public confessions as transitional structures from the paranoid, persecutory world characteristic of the old system of village life, in which it is possible to grasp both a latent demand for dependence and protection (which is one of the peculiar ways by which the soothsayer, the healer or householder manipulate social ties), to that of depressive guilt and individualization, which represents, properly speaking, the process of internalization of guilt and the ability to take on one's own aggression.

Another important contribution comes from Henry Murphy. Through a historical comparison between sixteenth‐century England and twentieth‐century Ghana, Murphy examined the role of three possible underlying factors. 1) Changes in the religious order, 2) Changes in the economic order and 3) changes in childhood educational practices (Murphy, 1995).

According to Murphy, it is primarily these changes that can explain the emergence of feelings of guilt, starting from the attitude of recognizing the source of experiences of pleasure or pain in one's own acts rather than as the result of chance, or of the actions of others.

Murphy’s perspective should be adopted also when looking at *our own cultures*, as it powerfully captures the element of structural change together with the complex psychological dynamics of those societies, too often described as timeless and ahistorical.

I would like to suggest an interpretation of the phenomenon of mass shooters that relates to Maurice Bloch’s anthropological theory of “rebounding violence” (1991). Bloch’s starting point is the archetypal, founding violent experience of being either predator or prey. From this universal, persecutory experience of having been for time immemorial prey animals in nature and constantly exposed to death, the issue at stake is how to transform one from being a prey of a savage nature, to becoming a culturalized hunter who may transcend death.

The essence of religion, for Bloch, as in Burkert (1983) or Girard (1977), is the archetypal violence which ignites the “transcendental” (as Bloch writes) ritual operation by which the human archetypal prey is transformed into a cultural hunter and at the same time a member of a specific symbolic society of hunters. Through this transformation, intra‐group social relations can be constructed, in which the members feel able to master their own existences before their own mortality and the archetypal, absolute violence of nature.

In his *Prey into Hunter* (1991), Bloch examines the practice of hunting and its symbolic importance among the Betsileo people of Madagascar. He explores how hunting is tied to social status and power dynamics. Hunting is portrayed as an activity that reinforces the hierarchical structures in the community, where hunters hold a privileged position. Bloch shows how violence, in this context, becomes a social and a cultural act, and not merely a “natural” means of survival. This archetypal circulation of savage violence needs an archetypal device to transform an original being (a child to be initiated) from an animal‐prey of wild nature into a full human‐hunter — a socialized adult belonging to a specific symbolic, social group.

Now, in line with his broader anthropological theories, Bloch examines how acts of violence — such as hunting and warfare — can have unintended consequences; how violence often "rebounds" in a way that reinforces social inequalities and can lead to a cyclical, destructive nature of in‐group conflict. The symbolic power that violence generates can lead to its perpetuation, often beyond the initial intention of those involved. In this perspective, the Betsileo initiation ritual not only transforms children‐prey into adult‐hunters but also provides a second function: that of containing and projecting into the out‐group the rebounding violence produced by this archetypal, oppositional structure made by prey and hunters. Bloch shows how this aggressive oral archetypal structure is present in the four corners of the world, from Africa to Japan, so that we may implicitly appreciate the importance of having effective rituals to perform such a transformation in viable ways.

In an orally greedy society, such as the American Society, in which the predominant belief is that all must be destined to be a winning hunter, entitled to consume (here meaning also “control”) everything as its own prey, accompanied by the de‐symbolizing absence of any ritual device, the stage is set for violence. A situation arises in which so many of the citizens, while expecting to be hunters, are anomically *destined* to be archetypal prey and ‐ due to the puritan Calvinist idea of double predestination ‐ feel this as their own shameful responsibility. This is a guilt too heavy to bear, which, in specific historical conditions, tilts into a paranoid reaction.

As Max Weber and Walter Benjamin understood, in the Western world we all miss a lost enchantment. Our hyper‐factual, naturalistic ontology has lost any proper ritual, symbol‐making device to metabolize our archetypally fragile and at the same time violent nature within the trophic chain that Descola mentioned in our first room, in which man (like any other animal) is a potential Prey to be consumed by a Hunter — to be annihilated *by* and *for* an alien potency.

From this perspective, this capitalist postliberal world seems the nightmarish distorted reproduction of the ancestral dangerous ancestral savanna of the hominids—not subjects *within* nature but subjects *to* nature—in which the only structural rule is *mors tua, vita mea*: your death, my life. This is a scenario that has become wholly unbalanced, which has lost the necessary symbolic device to contain and transform the archetypal fear of the human animal for which, at the end of the competitive, capitalist chain, just one, ultimate, last hunter would paradoxically survive—the one who would have consumed all of his prey.

Yet, we shall be reminded of what happened to Orion, the Archetypal Hunter, hunted down by Artemis — Nature itself. In this wild ontology, the struggle to preserve and increase one’s will to power — one’s feeling of agency, which is an essential aspect of individuation — is caught between the wheels of a totally unsettled machine. In the case of the contemporary USA, the symbolic crisis collapsed when the puritan myth was contradicted by the loss of the Vietnam War. This showed that there was no more physical and symbolic earthly space to conquer; no more space to consume in pursuit of American manifest destiny. This, for instance, prolonged the mythical quest for conquer and consumption of the original pioneer to the skies — as J.F. Kennedy did with the quest for the moon. Yet, this exhaustion of physical and mental space could not be transformed and it became increasingly difficult to project the shadow of the puritanic ideology of the *House on the Hill* into the space of the Other others — a projected impure, inferior shadow to be conquered and violently consumed (Carta, 2020).

My hypothesis is that these dynamics unleash, in an uncontrolled way, Bloch’s “rebounding violence” under the form of the mass shooters, who are unconsciously, blindly trying to re‐perform an archetypal ritual, now degenerated. The feeling of responsibility for not being up to the optimistic promise for which the Good Ones will win tilts responsibility and guilt into shame and can turn depression into paranoia.

In the capitalist de‐symbolized, anomic context, the proliferation of tattoos, piercings or psychotropic drugs is a sign of this unsatisfied archetypal need for the lost rituals of passage—for the transformation of nature (all nature, not just our nature) into culture. Today people tattoo their bodies, sometimes compulsively, with images that seem to proliferate without any transcendent meaning; or they pierce new openings as shamans do, yet without having any idea of why they wish to do it.

My supposition is that mass shooters are unconsciously staging a desperate, ultimate archetypal ritual in which, for a moment, they may turn from prey to hunters. Yet, if this hypothesis is right, this transformation would be a symptom in which nothing is really resolved. In the perspective of this symbolic failure, the fact that almost all mass shooters at the same time plan, expect and look for their own death would make very much sense. Caught in a “natural” —non culturalized—condition, they become the victims of their rebounding violence: they are at the same time hunters *and* prey, and are transformed by this tremendous archetypal force that no ritual can harness into hybrid, monstruous mythical creatures: half depressed, half paranoid.

Forget any behaviourism, any ego‐centered psychology aiming at an a‐critical adaptation, an elimination of all disturbing symptoms via the outrageous multiplication of pathological categories as has been happening in the DSMs. We, analysts, are among the last ones of a lost species. Our analytical setting is the anthropological extension of what humanity has always tried to do from times immemorial—to transform our *prima materia*, what Jung called the *Anthropos*—into a culturalized, social, conscious existence through rituals and the myths that, below the surface, guide our lives. This is the reason why we should accept this anthropological continuity.

Forget any Western psychotherapeutic exceptionalism and look at what the *Other others* did and still do; how they deal with the same archetypal issues with which we humans are all confronted. Within the bewildering history of our planet, we, analysts or patients — the difference is insignificant — deep down are all the same; as Pascal would have written (1958, para. 233, pp. 66–67) — Sailing frail, brief, through infinity, all on the same boat. This condition of essential fragility is why we need to learn from the *Others* how to preserve the spark of our agency and to be able to share it, along with our capacity to love — may they be Micronesians Ifaluks, African Yoruba or Wolof, North American Cree, Amerindian Nambiwkara or Aborigenal Warlpiri.

T.S. Eliot knew this fragile essence of ours well, when he wrote:

Go, go, go, said the bird: human kind

*Cannot bear very much reality*. 3

## Data Availability

The data that support the findings of this study are available on request from the corresponding author. The data are not publicly available due to privacy or ethical restrictions.

## Footnotes

^2^
See also the outstanding seminal paper by Viveiros de Castro, 2012.

^3^
Eliot, T. S. (1947). *Four Quartets. Burnt Norton*. Harcourt Brace and Company.
